# Integrated Physiological, Biomechanical, and Subjective Responses for the Selection of Assistive Level in Pedelec Cycling

**DOI:** 10.3389/fphys.2021.740728

**Published:** 2021-12-08

**Authors:** Sheng-Chieh Yang, Yun-Ju Lee

**Affiliations:** ^1^Cycling & Heath Tech Industry R&D Center, Taichung, Taiwan; ^2^Department of Industrial Engineering and Engineering Management, National Tsing Hua University, Hsinchu, Taiwan

**Keywords:** pedelec, simulated slope resistance, assistive power, physical intensity, muscle activity, rating of perceived exertion

## Abstract

In recent decade, pedelec has become one of the most popular transportation modes due to its effectiveness in reducing physical effort. The effects of using pedelec as an alternative mode of exercise were explored in previous studies. However, the effects of pedelec parameters were not quantified for the self-selected gear ratio, random riding speed, and varied road slopes, which restricted its application. Hence, this study aimed to evaluate the effects of gear ratio and assistive torque and to determine the optimum riding condition regarding physiological, biomechanical, and subjective responses of the rider. The riding tests consisted of simulated slope (1.0 vs. 2.5% grade), gear ratio (light vs. heavy), and assistive levels (0.5, 1, 1.5, and 2), and the tests were conducted in a randomized order. A total of 19 non-athletes completed the riding tests to evaluate physiological [metabolic equivalent of task (MET), heart rate, and gross efficiency (GE)], biomechanical [muscle activity (expressed as reference voluntary contraction, RVC) and power output], and subjective responses [rating of perceived exertion (RPE) and sense of comfort (SC)]. The test conditions induced moderate to vigorous intensities (3.7–7.4 METs, 58.5–80.3% of maximal heart rate, 11.1–29.5% of RVC rectus femoris activity, and 9.4–14.2 RPEs). The effects of gear ratio and assistive level on the physiological responses were significant. Riding with the heavy gear ratio showed advantages in METs and GE. For the optimum assistive level selection, low GE and limited improvement in subjective responses suggested the impact of low-power output conditions. Overall, for the health pedelec commuters, riding with 0.75 W/kg power output with 50 rpm cadence is recommended to obtain the moderate intensity (4.7 METs) and the advantages in GE and subjective feelings. Moreover, the findings can be applied to exercise intensity control and save battery energy effectively in varying riding conditions.

## Introduction

Active commuting by bike induces cardiorespiratory and muscular loads, which fulfills the required intensity and volume for maintaining health (Hendriksen et al., 2000; Oja et al., 2011; Schäfer et al., 2020). However, uphill the route would affect the motivation for active commuting due to the fear of vigorous-intensity (van Bekkum et al., 2011). Addressing the need for avoiding strenuous physical effort during commuting, pedelec, known as an electric-assisted bike, is gaining popularity for its effectiveness in providing an easier pedaling experience. The magnitude of assistive torque is provided proportionally to the pedaling torque of the rider, and an assistive level denotes the relative contribution of the motor and the rider. For example, with the assistive level of 0.5, the motor will generate 10 newton-meter (Nm) torque while the rider produces 20 Nm pedaling torque. The other measure to obtain the preferred pedaling torque and cadence is adjusting the gear ratio. Combining the manual adjustment of pedelec parameters, the pedelec riders could maintain the intensity level when the slope or speed changes. But there is still a gap in integrated evaluation of the effect of pedelec parameters, which leads to the limits in optimizing riding experience, progressing toward auto-adjusting, exercising prescription execution, and effectively managing battery energy.

The assistive torque of the pedelec helps overcome the challenges of prolonging riding, long distances, or uphill for untrained or sedentary people (Gojanovic et al., 2011; Sperlich et al., 2012). Previous studies have investigated the effects of assistive torque on physical intensity in flat and uphill route ridings and found that the intensities, as evaluated by the metabolic equivalent of task (MET), ranged from moderate (3–6 METs) to vigorous (>6 METs) levels (Simons et al., 2009; Gojanovic et al., 2011). Compared with the conventional bicycle or the pedelec without assistance, riding with the assistive torque resulted in significantly decreased oxygen consumption, energy expenditure, heart rate (Simons et al., 2009; Gojanovic et al., 2011; Louis et al., 2012; Langford et al., 2017), and muscle activity (Sperlich et al., 2012). Pedelec riding was also found to facilitate subjective feelings (Simons et al., 2009; Gojanovic et al., 2011; Louis et al., 2012; Sperlich et al., 2012; Langford et al., 2017). Previous studies reported the significantly lower rating of perceived exertion (RPE) (Simons et al., 2009; Gojanovic et al., 2011; Louis et al., 2012) with the simultaneously higher level of enjoyment (Langford et al., 2017) and sense of comfort (SC) (Simons et al., 2009). Moreover, for the moderate riding intensity, a pedelec is also thought to be a proper modality for commuting (Simons et al., 2009; Gojanovic et al., 2011) purposes. For the untrained or sedentary people, the health benefits of using pedelec transportation include improved maximal power output (De Geus et al., 2013), post-OGTT (oral glucose tolerance test), and maximum oxygen consumption (Peterman et al., 2016).

Although gear ratio adjustment was not documented explicitly in previous pedelec studies, it was evidenced by the simultaneous changes in pedaling rate and speed. In the studies that allowed the pedelec riders to choose their preferred speed with an assigned assistive level, the rider generally adopted a faster speed with a constant (Gojanovic et al., 2011) or decreased pedaling rate (Simons et al., 2009) compared with the non-assistance condition, which indicated a heavier gear ratio was selected. This might be explained by the gear-shifting behaviors of professional cyclists in racing events. Professional cyclists attempt to ride with the optimum pedal torque and pedal rate to minimize the physiological and biomechanical load (Chavarren and Calbet, 1999; Watson and Swensen, 2006; Abbiss et al., 2009) through the gear ratio adjustment. A light gear ratio that leads to the lower pedal torque is chosen to avoid the use of the less fatigue-resistance type II muscle fibers (Lucía et al., 2001). In contrast, a heavy gear ratio increases the required torque but reduces the pedal cadence for the desired speed and saves the bioenergy expenditure caused by the repetitive limb movement (Chavarren and Calbet, 1999; Louis et al., 2012). For the pedelec riders, using the heavier gear ratio under the assigned assistive level may imply the need for compensating the low resultant torque under the use of assistance. In contrast, this also indicated the motor generated excessive assistive torque and suggested the consequent battery energy wasting. From the health benefit and energy-saving points of view, the proper assistive level should be determined based on physiological, biomechanical, and subjective responses. However, there is still limited knowledge of effective pedelec parameter adjusting to maintain the preferred intensity while avoiding energy wasting.

The pedelec was a proper modality for exercise training (De Geus et al., 2013; Peterman et al., 2016) and commuting, but the non-integrated evaluations of assistive level and gear ratio under the varying slopes limit the improvement of training effectiveness or user satisfaction. A recent review reported two of the most prevalent barriers of pedelec riders, namely, less physical activity and range anxiety (Bourne et al., 2020), which imply the need for a comprehensive investigation of rider response and the required assistance. Both of the barriers are mainly associated with excessive assistive torque. Due to the random changes in slope and speed in previous studies, the data are inadequate to develop the strategy that is effectively keeping optimum physiological, biomechanical, and subjective status. Therefore, this study aimed to elicit a wide range of responses in the simulated riding conditions and investigate the responses, especially under low-power output conditions to identify the improper assistive level. The hypothesis was that the positive effect of torque assistance on rider responses is limited, particularly in the low-power output conditions. A series of indoor pedelec riding tests with simulated slope resistances were conducted to eliminate the random effects of varying inclination in outdoor riding.

## Materials and Methods

### Participants

A total of 10 female and 9 male healthy (without musculoskeletal and cardiorespiratory disorders) adults were enrolled in this study. The protocol was approved by the Ethics Committee of the National Tsing Hua University (REC: 10811HE094). The participants were given an introduction to the aim and procedure of the study. After fully understanding and being willing to join, the participants signed the informed consent. Before data collection sessions, the Chinese version of the “Physical Activity Readiness Questionnaire for Everyone” (PAR-Q+) (Warburton et al., 2018) survey was used to assess the readiness of the participant in performing the indoor riding test. All participants were free from disorders listed in the questionnaire.

Personal data, such as age, body height, and body mass, were recorded. Besides, self-reported physical activity status, frequency (number of times per week), duration, and type were also recorded *via* a questionnaire. According to the self-reported data, all the participants were non-athlete adults, and 14 participants engaged in recreational physical activities. All participants had experiences in riding conventional bikes.

### Experiment Procedure

The experiment consisted of 16 indoor pedelec riding tests. On the day of the riding test, the participants were asked to intake a meal at least 2 h before the riding test. Alcohol and caffeine were restricted. The saddle height was adjusted according to the inseam length of the participants. Before the riding test, the participants were familiarized with riding at the target riding speed, 21 km⋅h^–1^ (Louis et al., 2012; Boele-Vos et al., 2017) when two different gear ratios were used. The real-time speed and cadence feedback were displayed on a monitor in front of the rider. All participants were able to maintain the target speed within ± 1 km⋅h^–1^ by controlling their cadence. The participants were then warmed up with the resistance relative to the flat road (0% slope) for 5 min.

For each riding test, the simulated slope resistance was provided, while the gear ratio and assistive level were specified. The simulated slopes were 1 and 2.5%. With the target riding speed of 21 km⋅h^–1^, the heavy (H, 46:14) and light (L, 46:17) gear ratios resulted in the cadence of approximately 50 and 60 revolutions per minute (rpm) were chosen, respectively. Around 50 rpm was the cadence freely selected by the participants in previous outdoor studies (Simons et al., 2009), and 60 rpm was suggested to be the most efficient cadence (Gojanovic et al., 2011). As for the assistive levels, “0.5”, “1”, “1.5”, and “2” were selected in the experiment, where “0.5” denotes the motor provided half of the torque of the rider under the testing conditions. The conditions were conducted in a randomized order. For each condition, the participants continuously rode on the indoor pedelec for 3 min (De Koning et al., 2012; Bini et al., 2019), and 3 min of rest were provided between the test conditions. In the rest period, the participants were asked to stand beside the pedelec. Each test started with the participant resting their feet on the pedal and ended for 3 min. Physiological and biomechanical responses were recorded for the entire 3-min period, and the subjective responses were recorded at the end of each condition.

**FIGURE 1:**
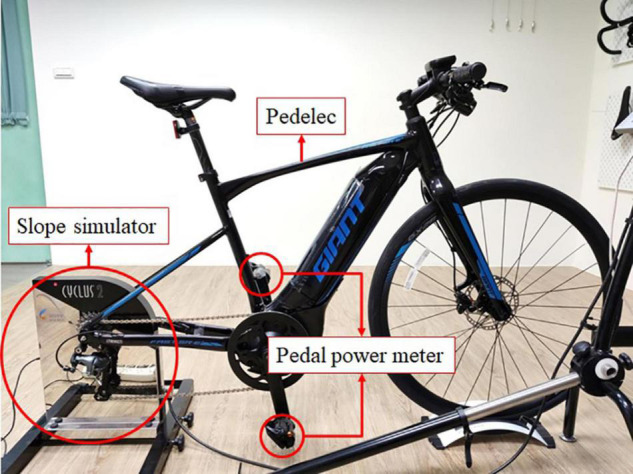
The pedelec, pedal power meter, and the slope simulator.

For the riding test, a pedelec with a motor located at the bracket bottom (Fast SR E+, Giant, Taiwan) was adopted. The original pedals of the pedelec were replaced by a pair of pedal power meters (PowerTap P1, SRAM, Chicago, IL, United States) (Pallarés and Lillo-Bevia, 2018; Wright et al., 2019) to measure the power output of the rider (watt, W) and pedal cadence (revolution per minute, rpm) (Figure 1). The validity and reliability of PowerTap P1 had been reported as acceptable (with rho >0.98 and mean CV = 2.3% compared with the gold standard) in the previous study (Pallarés and Lillo-Bevia, 2018). The pedal power meter was zero calibrated before riding tests. Once the speed attained 21 km⋅h^–1^, the power output and cadence data were utilized for analysis. For the simulated slope assistance generation, the rear wheel of the pedelec was removed and replaced by an indoor trainer (Cyclus2, RBM Elektronik-automation GmbH, Leipzig, Germany). The indoor trainer is capable of providing simulated slope resistance according to the target slope, the body mass of the rider and the front projection area (Debraux et al., 2011), the mass of the pedelec (16 kg), the riding speed, and the road surface coefficient of rolling resistance (Ba Hung et al., 2017).

### Measurement of Physiological Responses

The participants wore a face mask and a chest strap heart-rate monitor during the riding test. The face mask covers the nose and mouth of the participant and collects gas samples with a sampling tube and a flowmeter. Breathing gas and heart rate data were transmitted to a gas analyzer (Quark CPET, COSMED, Italy). Before data collection, the standard gas (16% oxygen and 5% carbon dioxide) was used to calibrate the gas analyzer, and a 3-L cylinder pump was used to calibrate the flowmeter. Energy expenditure (EE) was estimated *via* oxygen and carbon dioxide data (Simons et al., 2009). Breath-by-breath oxygen consumption (_$\dot{V}\,{\text{O}}_{2}$_, ml⋅kg^–1^⋅min^–1^), energy expenditure (Joule⋅min^–1^), and heart rate (beat per minute) data from the last 30-s period of each test were averaged for further analysis. Previous studies suggested moderate intensity (3–6 METs) as the appropriate intensity for commuters (Simons et al., 2009; Gojanovic et al., 2011; Louis et al., 2012; Berntsen et al., 2017), and the criteria can be defined accordingly in this study.

### Measurement of Biomechanical Responses

Muscle activity of rectus femoris (RF), vastus lateralis (VL), and vastus medialis (VM) of the dominant leg (determined by asking the participant to kick an object with the intuitively selected leg) were measured and analyzed using wireless surface electromyography (EMG) sensors (TeleMyo DTS, Noraxon, Scottsdale, AZ, United States) and the MyoResearch software (version 3.16.32, Noraxon, Scottsdale, AZ). The parameters of the EMG sensor were as follows: baseline noise <1 μV RMS; input impedance >100 Mohm; CMR >100 dB; and overall gain = 200. Skin preparation was done before the placement of the electrodes. The locations of the electrode were determined according to the recommendation from SENIAM (Hermens et al., 2000).

### Measurement of Subjective Responses

The subjective evaluation of exertion and SC was recorded at the end of each riding test. The RPE (Borg, 1982) was subjectively evaluated using the Borg scale (from 6, easy, to 20, maximal effort). The five-point Likert scale was used to evaluate the SC, where “1” represents the most comfort level and “5” represents the lowest comfort level (Simons et al., 2009). According to Simons et al. (2009), the criteria of RPE and SC can be defined as 13.1 and 1.7, respectively, to present the lower limits of subjective responses when assistance was not provided. Namely, once the assistance is provided, the score of RPE and SC should be lower than the reference value as the assistive level increases.

### Data Processing and Statistical Analysis

The MET and normalized heart rate (nHR) were obtained from the physiological data to determine the relative intensities. The MET was obtained by dividing _$\dot{V}\,{\text{O}}_{2}$_ by 3.5 ml⋅kg^–1^⋅min ^–1^ (Simons et al., 2009; Louis et al., 2012). The nHR was obtained by dividing the mean heart rate by the age-predicted maximal heart rate (220 – age) for each participant (Garber et al., 2011). The processed data of each test were then categorized into one of the intensity levels from very light to maximal according to the position stand of the American College of Sports Medicine (Garber et al., 2011). For gross efficiency (GE) estimation, power output and EE data of the participant were used in the followed equation (Louis et al., 2012).


(1) $$\mathrm{Gross}\mathrm{efficiency}\left(\mathrm{GE},\%\right)=\frac{\mathrm{Work}\,\left(W,\mathrm{Joule}\right)}{\mathrm{Energy}\,\mathrm{Expenditure}\,\left(E,\mathrm{Joule}\right)}\times 100\%$$

The EMG data were filtered by the Butterworth bandpass filter (20–500 Hz) and smoothed *via* the root mean square (RMS) technique with a 50-ms window. Averaged EMG data from the last 10 crank cycles of each test were used for further analysis. An accelerometer (Accelerometer Wireless, Noraxon, Scottsdale, AZ, United States) was attached to the right crank with its orientation aligned with the crank stem to determine the individual crank cycle. The acceleration data were synchronized with EMG data, and the peak value represented the pedal located at the top position of the whole crank cycle. For the non-athlete participants, the reference voluntary contraction (RVC) method was used to normalize the EMG data for each muscle (Candotti et al., 2009; Sinclair et al., 2015). The maximum muscle activity level was assumed to be elicited from the highest muscle force demanded condition (i.e., 2.5% slope, heavy gear ratio, and assistive level of 0.5), and the EMG data were selected as the RVC.

All data were expressed as means ± standard deviation. Analyses were performed using SPSS version 17.0 (SPSS Inc, Chicago, IL, United States). As the assistive level is defined as a specific proportion of assistive torque to the pedal torque of the rider, the absolute assistive torque would be greater in high pedal-torque demand conditions even though the same assistance level is selected, which led to the complexity in data interpreting. Hence, data from different slope conditions were divided into 1.0% slope or 2.5% slope sets and analyzed, respectively. Considering the small size and the distribution normality of samples, generalized estimating equations (GEE) analyses were conducted using the factors of gear ratio [light (46:17) and heavy (46:14)] and assistance level (0.5, 1, 1.5, and 2). The condition with light gear ratio and assistive level of 2 served as the reference for all analysis. *Post hoc* pairwise comparisons were performed using the Bonferroni method. For the statistical analysis, a value of *p* < 0.05 was accepted as the level of significance.

## Results

### Characteristics of Participants

All participants completed the laboratory test. The mean age of the participant was 29.1 ± 6.2 years. Their body weight and body height were 65.6 ± 12.3 kg and 165.3 ± 8.0 cm, respectively. The mean physical activity frequency was 2.5 ± 2.0 times per week. Five participants did not have regular physical activity, and the other participants engaged in various habitual recreational activities.

### Effects of Gear Ratio and Assistance on Physiological Responses

In the 1.0% slope conditions, the effect of gear ratio was significant on METs (*p* = 0.001) and GE (*p* = 0.002). Significant effects of assistance were shown on METs, nHR (except for the assistive level of 1.5, *p* = 0.06), and GE. Significant interaction effects of gear ratio and assistance were revealed on METs and nHR only for the heavy gear ratio and assistive level of 1.5 conditions (Table 1). The result of pairwise comparison showed significant differences between assistive levels (level 0.5 > 1 > 1.5 > 2, all *p* < 0.01) in METs, nHR, and GE, respectively.

**TABLE 1:** Coefficients (β), standard errors (S.E.) and p values of physiological responses in 1.0% slope conditions.

	METs			nHR			GE		
	β	S.E.	*p*	β	S.E.	*p*	β	S.E.	*p*
Intercept	4.87	(0.13)	<0.001	63.95	(2.04)	<0.001	13.72	(0.37)	<0.001
**G**									
Heavy	−0.15	(0.05)	0.001	−0.15	(0.71)	0.267	0.52	(0.21)	0.002
**A**									
0.5	2.51	(0.11)	<0.001	16.39	(1.50)	<0.001	4.82	(0.26)	<0.001
1	1.37	(0.10)	<0.001	9.46	(1.24)	<0.001	3.31	(0.22)	<0.001
1.5	0.57	(0.08)	<0.001	4.20	(1.07)	<0.001	1.63	(0.29)	<0.001
**G × A**									
H × 0.5	0.18	(0.10)	0.082	−0.42	(1.05)	0.685	−0.51	(0.32)	0.115
H × 1	0.01	(0.08)	0.877	−0.74	(0.92)	0.419	−0.30	(0.30)	0.318
H × 1.5	0.06	(0.10)	0.520	0.08	(0.80)	0.921	−0.31	(0.33)	0.345

*The condition with light gear ratio (G) and assistive level (A) of 2 served as reference.*

**FIGURE 2:**
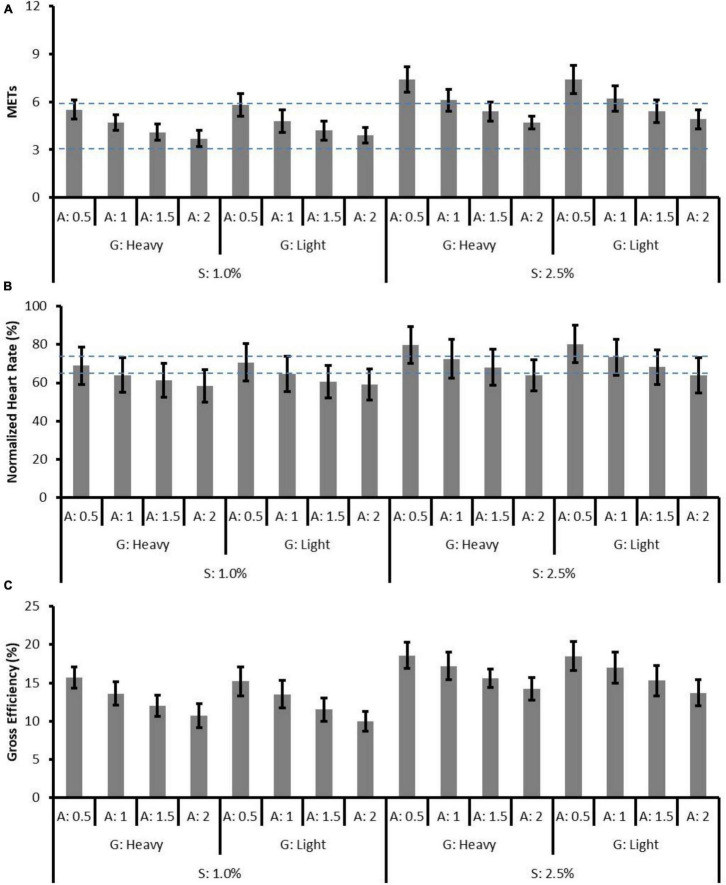
The means and standard deviations of **(A)** MET, **(B)** normalized heart rate (%), and **(C)** gross efficiency (%) in all conditions. S, slope; G, gear ratio; A, assistive level. The horizontal dashed lines represent the upper and lower bound of moderate level defined by ACSM.

In the 2.5% slope conditions, the effects of gear ratio on MET (*p* = 0.004) and GE (*p* = 0.014) were significant. The effect of assistance was significant on METs, nHR, and GE (with all *p* < 0.01), whereas the interaction effects of gear ratio and assistance were not significant (Table 2). Likewise, the results of pairwise comparison showed significant differences between assistive levels in METs, nHR, and GE (level 0.5 > 1 > 1.5 > 2, all *p* < 0.01). Tables 1, 2 present the coefficients (β), standard errors, and *p* values of physiological responses in 1.0 and 2.5% slope conditions, respectively. Figures 2A–C depicts the mean MET, nHR, and GE of each riding condition. According to ACSM, the mean METs corresponded with moderate to vigorous levels, respectively, the mean nHRs corresponded with light to vigorous levels.

**TABLE 2:** Coefficients (β), standard errors (S.E.) and p values of physiological responses in 2.5% slope conditions.

	METs			nHR			GE		
	β	S.E.	*p*	β	S.E.	*p*	β	S.E.	*p*
Intercept	3.88	(0.11)	<0.001	59.08	(1.83)	<0.001	9.99	(0.30)	<0.001
**G**									
Heavy	−0.20	(0.06)	0.001	−0.61	(0.55)	0.267	0.66	(0.22)	0.002
**A**									
0.5	1.89	(0.07)	<0.001	11.64	(1.18)	<0.001	5.22	(0.32)	<0.001
1	0.88	(0.09)	<0.001	5.54	(0.95)	<0.001	3.51	(0.33)	<0.001
1.5	0.30	(0.08)	<0.001	1.41	(0.75)	0.059	1.53	(0.30)	<0.001
**G × A**									
H × 0.5	−0.06	(0.11)	0.617	−1.26	(0.67)	0.061	−0.22	(0.38)	0.557
H × 1	0.13	(0.11)	0.236	0.02	(0.79)	0.979	−0.53	(0.37)	0.152
H × 1.5	0.17	(0.08)	0.035	1.41	(0.7)	0.044	−0.21	(0.25)	0.399

*The condition with light gear ratio (G) and assistive level (A) of 2 served as reference. METs: metabolic equivalent of task. nHR: normalized heart rate, %. GE: gross efficiency, %.*

### Effects of Gear Ratio and Assistance on Biomechanical Responses

In the 1.0% slope conditions, the effect of gear ratio was not significant on RF, VL, and VM activities. The effects of assistance were significant on RF (except for assistive level of 1.5, *p* = 0.12), VL (except for assistive level of 1.5, *p* = 0.31), and VM (except for assistive level of 1.5, *p* = 0.07). There was no interaction effect found on the muscles. The results of pairwise comparison revealed significant activity differences between the assistive levels (assistive level of 0.5 > 1 > 1.5 > 2, all *p* < 0.01) in each muscle.

**FIGURE 3:**
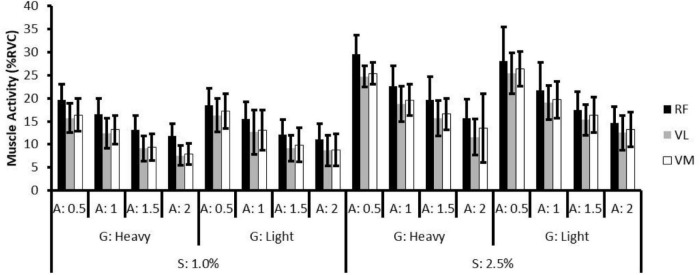
The mean and standard deviation muscle activity of rectus femoris (RF), vastus lateralis (VL), and vastus medialis (VM) in all conditions. S, slope; G, gear ratio; A, assistive level.

In the 2.5% slope conditions, the effect of gear ratio was not significant on RF, VL, and VM activities, whereas the effects of assistance were significant on RF, VL, and VM (all *p* < 0.01). No significant interaction effect was found in each muscle. The results of pairwise comparison revealed significant activity differences between the assistive levels (level of 0.5 > 1 > 1.5 > 2, all *p* < 0.01) in each muscle. Tables 3, 4 present the coefficients (β), standard errors, and *p* values of biomechanical responses in 1.0 and 2.5% slope conditions, respectively. Figure 3 depicts the muscle activity of each riding condition.

**TABLE 3:** Coefficients (β), standard errors (SE), and *p*-values of biomechanics responses in 1.0% slope conditions.

	RF			VL			VM		
	β	SE	*p*	β	SE	*p*	β	SE	*p*
Intercept	11.14	(0.75)	<0.001	8.7	(0.73)	<0.001	8.83	(0.76)	<0.001
**G**									
Heavy	0.61	(0.69)	0.374	−1.13	(0.63)	0.074	−0.98	(0.58)	0.093
**A**									
0.5	7.39	(0.59)	<0.001	7.55	(0.49)	<0.001	8.37	(0.51)	<0.001
1	4.35	(0.7)	<0.001	4.01	(0.75)	<0.001	4.24	(0.73)	<0.001
1.5	0.93	(0.59)	0.116	0.51	(0.51)	0.313	1.06	(0.58)	0.067
**G × A**									
H × 0.5	0.43	(0.71)	0.543	0.58	(0.79)	0.464	0.16	(0.73)	0.825
H × 1	0.42	(0.7)	0.549	0.8	(0.67)	0.234	1.11	(0.73)	0.128
H × 1.5	0.56	(0.73)	0.442	1.07	(0.85)	0.207	0.52	(0.8)	0.516

*The condition with light gear ratio (G) and assistive level (A) of 2 served as reference. RF, muscle activity of rectus femoris, %RVC; VL, muscle activity of vastus lateralis, %RVC; VM, muscle activity of vastus medialis, %RVC.*

**TABLE 4:** Coefficients (β), standard errors (SE), and *p*-values of biomechanics responses in 2.5% slope conditions.

	RF			VL			VM		
	β	SE	*p*	β	SE	*p*	β	SE	*p*
Intercept	14.69	(0.78)	<0.001	12.51	(0.85)	<0.001	13.17	(0.85)	<0.001
**G**									
Heavy	0.9	(0.87)	0.374	−0.94	(0.86)	0.074	0.32	(1.76)	0.093
**A**									
0.5	13.43	(1.68)	<0.001	12.85	(0.63)	<0.001	13.19	(0.65)	<0.001
1	6.96	(1.27)	<0.001	6.49	(0.58)	<0.001	6.57	(0.66)	<0.001
1.5	2.83	(0.71)	<0.001	2.78	(0.58)	<0.001	3.19	(0.61)	<0.001
**G × A**									
H × 0.5	0.46	(1.36)	0.732	0.32	(0.8)	0.69	−1.28	(1.69)	0.448
H × 1	0.04	(1.03)	0.969	0.75	(1)	0.456	−0.43	(1.74)	0.806
H × 1.5	1.14	(1.03)	0.272	1.37	(1.03)	0.184	−0.07	(1.77)	0.969

*The condition with light gear ratio (G) and assistive level (A) of 2 served as reference.*

**FIGURE 4:**
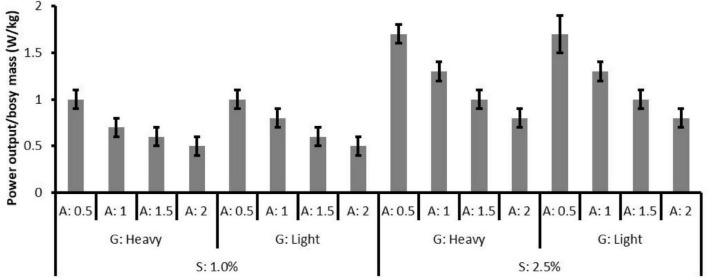
The mean and standard deviation of normalized power output (W/kg) in all conditions.

In both 1.0 and 2.5% slope conditions, the effects of gear ratio on power output were not significant (*p* = 0.85 and 0.91), while the effect of assistance was significant (both *p* < 0.01). The results of pairwise comparison indicated significant differences between assistive levels (Figure 4) (level of 0.5 > 1 > 1.5 > 2, all *p* < 0.01). The non-significant effect of gear ratio on power output (*p* = 0.88) indicated that in the same slope and assistance conditions, the participants could maintain the same power output with the heavy and light gear ratio settings, i.e., the pedal cadence and pedal torque may account for the differences in the measured responses.

### Effects of Gear Ratio and Assistance on Subjective Responses

In the 1.0% slope conditions, the effect of gear ratio was not significant on RPE (*p* = 0.85) and SC (*p* = 0.56). The effect of assistance on RPE was significant only with the assistive level of 0.5 (*p* = 0.016) but not significant on SC. Significant pairwise RPE differences were found between the assistive level of 0.5 and the other levels, respectively, whereas no significant pairwise difference was found between assistive levels of 1, 1.5, and 2 (level 0.5 < 1 = 1.5 = 2). As for SC, the only pairwise difference was found between the assistive level of 1.5 and 2 (1.66 ± 0.14 vs. 2.05 ± 0.20, *p* = 0.017).

**FIGURE 5:**
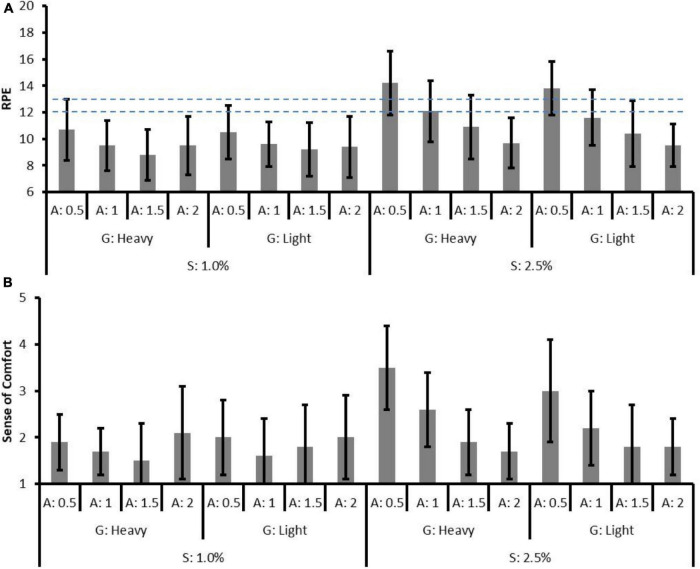
The mean and standard deviation of **(A)** rate of perceived exertion (RPE) and **(B)** sense of comfort (SC) in all conditions. The horizontal dashed lines represent the upper and lower bound of moderate level defined by ACSM.

Tables 5, 6 present the coefficients (β), standard errors, and *p* values of subjective responses in 1.0 and 2.5% slope conditions, respectively. Figures 5A,B depicts the results of RPE and SC. In the 2.5% slope conditions, the effect of gear ratio was not significant on RPE (*p* = 0.40) and SC (*p* = 0.36). The effect of assistance was significant on RPE and SC (except for assistive level of 1.5). Significant pairwise RPE differences were found between each assistive levels (level 0.5 > 1 > 1.5 > 2, all *p* < 0.01), while pairwise SC differences were found between each assistive level, except for levels 1.5 and 2 (level 0.5 > 1 > 1.5 = 2).

**TABLE 5:** Coefficients (β), standard errors (SE), and *p*-values of subjective responses in 1.0% slope conditions.

	RPE			SC		
	β	SE	*p*	β	SE	*p*
Intercept	9.42	(0.51)	<0.001	2	(0.2)	<0.001
**G**						
Heavy	0.05	(0.28)	0.853	0.11	(0.18)	0.56
**A**						
0.5	1.11	(0.46)	0.016	0	(0.24)	1
1	0.16	(0.49)	0.747	−0.42	(0.25)	0.093
1.5	−0.21	(0.25)	0.406	−0.21	(0.16)	0.186
**G × A**						
H × 0.5	0.11	(0.46)	0.821	−0.16	(0.2)	0.431
H × 1	−0.16	(0.41)	0.7	0.05	(0.32)	0.869
H × 1.5	−0.42	(0.25)	0.093	−0.37	(0.33)	0.259

*The condition with light gear ratio (G) and assistive level (A) of 2 served as reference.*

*RPE: rating of perceived exertion.*

**TABLE 6:** Coefficients (β), standard errors (SE), and *p*-values of subjective responses in 2.5% slope conditions.

	RPE			SC		
	β	SE	*p*	β	SE	*p*
Intercept	9.47	(0.37)	<0.001	1.84	(0.13)	<0.001
**G**						
Heavy	0.26	(0.31)	0.853	−0.16	(0.17)	0.56
**A**						
0.5	4.32	(0.41)	<0.001	1.16	(0.28)	<0.001
1	2.16	(0.37)	<0.001	0.37	(0.19)	0.093
1.5	0.89	(0.36)	0.406	0	(0.2)	0.186
**G × A**						
H × 0.5	0.16	(0.39)	0.685	0.68	(0.21)	0.001
H × 1	0.16	(0.58)	0.786	0.58	(0.25)	0.021
H × 1.5	0.32	(0.56)	0.571	0.26	(0.27)	0.324

*The condition with light gear ratio (G) and assistive level (A) of 2 served as reference.*

## Discussion

**FIGURE 6:**
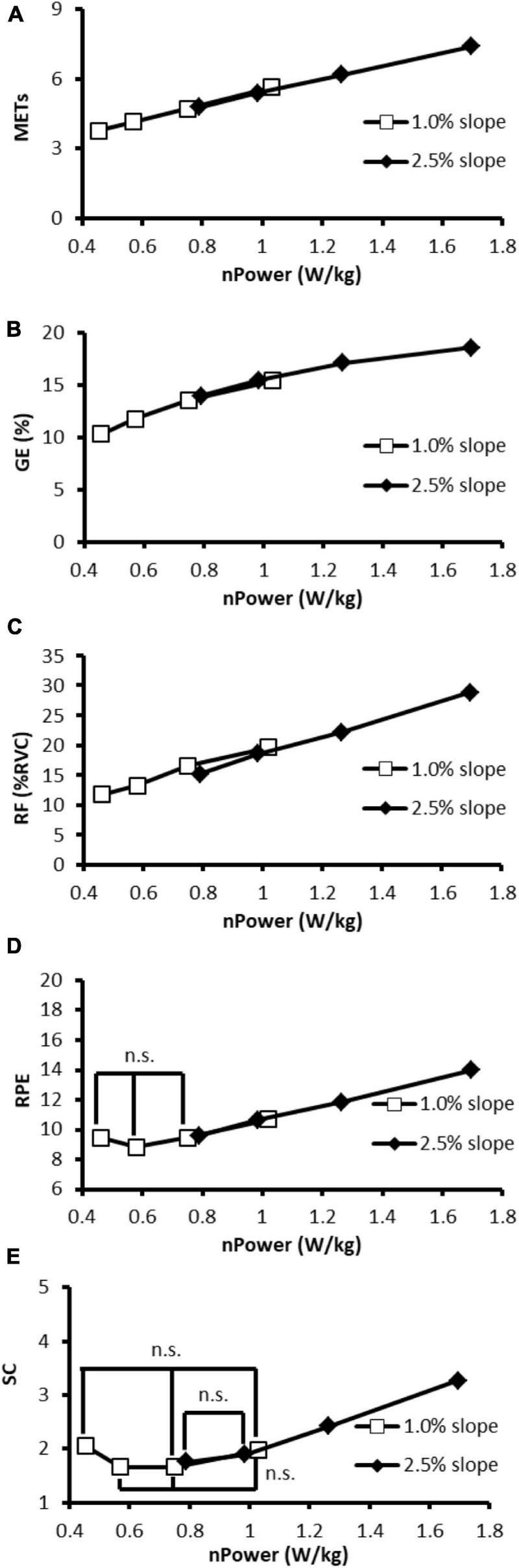
The relationship between **(A)** MET, **(B)** GE, **(C)** RF, **(D)** RPE, **(E)** SC, and the normalized power output. Data from heavy and light gear ratios are averaged. Pairwise differences existed between assistive levels except for the non-significant (n.s.) marked conditions. nHR, VL, and VM were omitted due to the similar patterns were presented.

This study investigated the effect of simulated slope resistance, gear ratio, and quantified assistive level on physiological, biomechanical, and subjective responses. In both slope conditions, the significant effects of gear ratio and assistance on physiological, biomechanical, and subjective responses were explored. The heavy gear ratio was associated with slight but significant lower METs and higher GE whereas did not influence the biomechanics and subjective responses. Increased assistive levels alleviated the METs, nHR, muscle activities and improved subjective responses but decreased GE. Furthermore, pairwise comparison between assistive levels of 1.5 and 2 in 1.0% slope conditions revealed that although there was a significant decrease in physiological load and muscle activity, limited improvement in subjective response might indicate the redundancy of assistive torque (Figures 6A–E).

### Pedelec Parameters and the Integrated Responses

The pedelec parameters resulted in moderate to vigorous intensity levels (3.7 to 7.4 MET) within the simulated slope conditions in this study. Although comparison among previous studies was difficult due to the differences in pedelec model and route, Gojanovic and colleagues (Gojanovic et al., 2011) reported 7.3 METs on a hilly route (with an average grade of 3.4%) with the “standard” assistance mode, and Simons and colleagues (Simons et al., 2009) reported 5.2 to 5.7 METs with “POW” and “ECO” mode on a flat route. The similar ranges of intensity would suggest the feasibility of slope simulation in this study. Likewise, despite the assistive power and the power output of the rider being rarely reported in the literature (Bulthuis et al., 2020), the assistive levels resulted in the averaged intensities of moderate and vigorous levels while no light level was shown, which also agreed with previous studies. This may further support the feasibility of the simulated pedelec riding as a standard test to evaluate the influences of a pedelec.

In Figure 6, the outcome measures are plotted against the normalized power output and the linear trends are presented in 2.5% slope conditions (black scatters). The power output changes due to assistive levels might explain the changes in physiological responses, muscle activity, and subjective responses. In 2.5% slope conditions, the lack of SC difference between assistive levels of 1.5 and 2 (Figure 5A) might be explained by few differences in power output. Taking a 70 kg adult as an example, the power output differences were only 14 W (the absolute power output with the assistive level of 1.5 is 70 kg × 0.99 W/kg = 69.3 W and with the assistive level of 2 is 70 kg × 0.79 W/kg = 55.3 W, respectively). Indeed, the15 W difference, as reported by Louis et al. (2012), was associated with no significant RPE difference in untrained participants. Similar results can be found in 1.0% slope conditions while assistive level of 0.5 (that resulted in 70 kg × 1.02 W/kg = 71.4 W power output) and assistive level of 1 (70 kg × 0.75 W/kg = 52.5 W) were used. Furthermore, despite the significant decreases in METs, nHR, and muscle activities were presented as the assistive level increased, RPE and SC showed a non-linear trend in 1.0% slope conditions. In the low-power output conditions (e.g., 1.0% slope, assistive levels of 1, 1.5, and 2), a non-significant decrease in RPE indicated that the assistive torque no longer generates subjective benefits. Moreover, the significantly worsen SC [increase in scores from 1.66 ± 0.14 (assistive level of 1.5) to 2.05 ± 0.2 (level 2), pairwise *p* = 0.017, Figure 6] might reflect the negative impact of high assistance in the low pedal-torque demand conditions. The limited subjective improvement in this study could be partly explained by the simultaneous decreases in GE (Figure 6). In this study, the GE increased with the power output and a positive relationship can be observed as found in professional cyclists (Chavarren and Calbet, 1999). In reverse, a systematic GE decreased (from 17.8 to 12.0%) with the decreased power output under different levels of assistance. The lower GE in the low-power output demand conditions might suggest that bioenergy was still consumed elsewhere (e.g., moving limbs) rather than generating power. Indeed, some of the participants verbally reported that excessive assistance caused difficulties in keeping the specific riding speed, and this might support the corresponding non-significant changes in the subjective response between assistive levels of 1, 1.5, and 2.

Using the heavy gear ratio induced a slight but statistically significant decrease in METs and an increase in GE. This finding might explain the adoption of the heavier gear ratio in previous studies (Simons et al., 2009; Gojanovic et al., 2011). Theoretically, using the light gear ratio decreased the required pedal-torque whereas it increased the required pedal cadence to maintain the same speed (10 more revolutions per minute). Although the muscle activity was not significantly affected by the differences in the required pedal-torque, the increased need for cadence might be the cause of the slight but significant increases in MET and decreases in GE. This might indicate that only a small portion of bioenergy was saved due to the decreased torque, but a relatively larger portion of bioenergy was still consumed in performing the additional limb movements. Meanwhile, muscular co-activation, frictions/viscous resistance of the joint cartilage, the ligaments, and the tendons may account for the slightly higher MET (Chavarren and Calbet, 1999; Louis et al., 2012).

### Pedelec Parameter Selection and Riding Intensity Control

Previous studies have recognized the health benefits of pedelec riding, and moderate exercise intensity has been suggested to ensure the benefits. The lowest intensity (3.7 METs) as found in the condition of 1.0% slope and assistive level of 2, fulfills the requirement of moderate-intensity (3 METs), and it is predictable that greater assistive levels would further decrease physiological and biomechanical intensity according to the linear relationship in Figure 6. However, the larger battery capacity and the greater electric assistance would annihilate the health-improving characteristics of pedelec riding (Gojanovic et al., 2011). Moreover, the limited effect and the negative effect of assistance in RPE and SC are foreseeable according to the findings from 1.0% slope conditions. Overall, moderate intensity can be achieved *via* pedelec parameter adjustments on different slopes. Simons et al. (2009) recommended that commuters use adequate assistance to prevents sweating on their way to work whereas use less assistance on the way home to gain training benefits. From the present findings, a more detailed suggestion can be made based on the integrated evaluation: choosing the pedelec parameters that result in about 50 rpm cadence and at least 0.75 W/kg power output to obtain physiological advantages and the acceptable subjective feeling while preventing battery energy wasting.

These findings can be applied to various fields without the constraints of specific pedelec models or test conditions. The exercise intensity could be controlled by changing the gear ratio and assistive level for the riders (Bulthuis et al., 2020). Based on Figure 6, the concept of intensity control can be supported by the overlapped portion of the two linear relationships of 1.0 and 2.5% slopes in which similar physiological, biomechanical, and subjective responses can be observed. It suggested the possibility of automatic assistance adjustment and exercise intensity control *via* a real-time algorithm to simplify pedelec operating and to achieve the fitness goals effectively. Future studies could address the assistance demand for the unfit, elderly, or disordered populations, whose demands may differ from the commuters. Moreover, establishing a personalized assistance control strategy that best meets the demands of an individual in various situations would be beneficial to the field.

### Limitations

Although the full-factorial experiment design is ideal for assessing the effects of all related factors thoroughly, several factors were not involved due to a considerable amount of tests that would be combined in testing limitations. For example, the effect of pedelec weight was not evaluated due to relatively less influence in slope resistance and rolling resistance, and a representative value, 16 kg, was adopted. Furthermore, the riding speed was not manipulated in this study, but the riding speed of 21 km⋅h^–1^ was specified to make our result comparable to the previous studies (Louis et al., 2012; Boele-Vos et al., 2017). The other limitation is the possible effect of fatigue in the high-power output demand conditions. Although the EMG data from the last 10 cycles enabled the comparison among the test conditions, the evaluation of fatigue within each test was not available. The changes in muscle activity in each condition might also provide important information that is related to the optimization of user experience.

## Conclusion

We proposed an integrated evaluation method that enables the analysis of the responses under various riding conditions. The effects of simulated slope, gear ratio, and numeric assistive level affected power output and the consequent physiological, biomechanical, and subjective responses. The pedelec parameters and the simulated slope resistance resulted in moderate to vigorous METs. In the low power, output demand conditions, increased assistive levels significantly decreased the MET, nHR, and muscle activities without improving the subjective feelings. For the pedelec commuters, riding with at least 0.75 W/kg resultant power output with about 50 rpm cadence is recommended to obtain the moderate intensity and the optimum subjective feelings. It is worth mentioning that preventing battery energy-wasting and releasing range anxiety might be the additional benefits of the recommended parameters.

## Data Availability Statement

The raw data supporting the conclusions of this article will be made available by the authors, without undue reservation.

## Ethics Statement

The studies involving human participants were reviewed and approved by the Ethics Committee of the National Tsing Hua University (REC: 10811HE094). The patients/participants provided their written informed consent to participate in this study.

## Author Contributions

S-CY and Y-JL conceived of the presented idea. S-CY carried out the experiment. S-CY wrote the manuscript with support from Y-JL. Y-JL supervised the findings of this work and edited the final manuscript. The authors discussed the results and contributed to the final manuscript. All authors contributed to the article and approved the submitted version.

## Conflict of Interest

The authors declare that no financial and personal relationships with other people or organizations have inappropriately influenced the content of the work reported in this manuscript.

## Publisher’s Note

All claims expressed in this article are solely those of the authors and do not necessarily represent those of their affiliated organizations, or those of the publisher, the editors and the reviewers. Any product that may be evaluated in this article, or claim that may be made by its manufacturer, is not guaranteed or endorsed by the publisher.
